# Genomic Insights Into the Interspecific Diversity and Evolution of *Mobiluncus*, a Pathogen Associated With Bacterial Vaginosis

**DOI:** 10.3389/fmicb.2022.939406

**Published:** 2022-07-05

**Authors:** Yisong Li, Ying Wang, Jie Liu

**Affiliations:** School of Public Health, Qingdao University, Qingdao, China

**Keywords:** *Mobiluncus*, comparative genomics, interspecific divergence, pathogenicity, horizontal gene transfer, bacterial vaginosis

## Abstract

Bacterial vaginosis (BV) is a common vaginal infection and has been associated with increased risk for a wide array of health issues. BV is linked with a variety of heterogeneous pathogenic anaerobic bacteria, among which *Mobiluncus* is strongly associated with BV diagnosis. However, their genetic features, pathogenicity, interspecific diversity, and evolutionary characters have not been illustrated at genomic level. The current study performed phylogenomic and comparative genomic analyses of *Mobiluncus*. Phylogenomic analyses revealed remarkable phylogenetic distinctions among different species. Compared with *M. curtisii, M. mulieris* had a larger genome and pangenome size with more insertion sequences but less CRISPR-Cas systems. In addition, these two species were diverse in profile of virulence factors, but harbored similar antibiotic resistance genes. Statistically different functional genome profiles between strains from the two species were determined, as well as correlations of some functional genes/pathways with putative pathogenicity. We also showed that high levels of horizontal gene transfer might be an important strategy for species diversification and pathogenicity. Collectively, this study provides the first genome sequence level description of *Mobiluncus*, and may shed light on its virulence/pathogenicity, functional diversification, and evolutionary dynamics. Our study could facilitate the further investigations of this important pathogen, and might improve the future treatment of BV.

## Introduction

Bacterial vaginosis (BV), a common gynecological disease characterized by vaginal discharge, affects roughly a quarter of women worldwide and costs an estimated $4.8 billion annually (Sobel, 2000; Javed et al., 2019; Peebles et al., 2019). BV has been associated with an increased risk of various health problems, including sexually transmitted infections, adverse pregnancy outcomes (e.g., preterm births, premature rupture of membranes), pelvic inflammatory disease, increased susceptibility to HIV infection, and other chronic health issues (Taha et al., 1998; Schwebke and Desmond, 2005; Onderdonk et al., 2016). The pathogenesis of BV is still a subject of debate (Cherpes et al., 2008; Muzny and Schwebke, 2016), whereas it has been determined that BV is characterized by a decrease in the levels of *Lactobacilli* and an overgrowth of opportunistic bacteria including anaerobes or microaerophiles such as *Prevotella, Gardnerella*, and *Mobiluncus* genera (Hillier, 1993; Sha et al., 2005). Among these, the abundance of Gram-negatively stained and curved rod-shaped bacteria, represented by *Mobiluncus*, has been considered as one of the key indicators of Nugent score, the “gold standard” for BV diagnosis (Nugent et al., 1991; Srinivasan et al., 2013).

*Mobiluncus* organisms were initially recognized in vaginal fluid as early as 1895 and were first isolated in 1913 (Curtis, 1913). Nowadays, much interest has revolved around *Mobiluncus* since women with higher Nugent scores (predominantly because of morphotypes consistent with *Mobiluncus*) are more likely to fail therapy than those with lower scores (Schwebke and Desmond, 2007), and the presence and persistence of *Mobiluncus* spp. was found to be highly associated with recurrence of BV (Meltzer et al., 2008). In addition, the production of malic acid and trimethylamine by *Mobiluncus* strains have been reported to give rise to vaginal irritation and unpleasant odor (Africa et al., 2014). For these reasons, more efforts have been made to characterize their resistance mechanisms and virulence factors (Spiegel, 1987; Zeng et al., 2020; Zhang et al., 2020). Nevertheless, the definite role of *Mobiluncus* in BV pathogenesis still remains largely elusive.

Analysis of the 16S rRNA gene sequences has revealed that *Mobiluncus* genus mainly contains two distantly related species, *M. curtisii* and *M. mulieris*. Previous reports from a number of laboratories have shown that these two species can be differentiated based on physical and biochemical properties. It has been demonstrated that *M. curtisii* and *M. mulieris* comprise short curved and long straight (or slightly curved) rods (Hoyles et al., 2004; Onderdonk et al., 2016), respectively, and show variation in growth in different liquid media (Taylor-Robinson and Taylor-Robinson, 2002). In addition, antigenic profiles of the two species are also distinct (Roberts et al., 1985; Gatti et al., 1997), and *M. mulieris* can stimulate a TLR5-mediated response in host, while *M. curtisii* cannot (Dela Cruz et al., 2021). Antimicrobial susceptibility and clonality of *Mobiluncus* also vary widely among species and even strains (Spiegel, 1987; Zhang et al., 2020). Recently, a new species collected from pig gut, namely *M. porci*, has been described (Wylensek et al., 2020). However, these studies were mostly based on phenotypic data, while genomic features, including genetic diversity and evolutionary history, of/between *Mobiluncus* species have not been clearly elucidated yet.

In the present study, we carried out an in-depth comparative genomic analysis of 38 publicly available genomic sequences of *Mobiluncus*, aiming to investigate the genomic diversity of this taxon. We compared the status of various virulence and antibiotic resistance genes (ARGs) among the strains in order to unleash the potential underlying mechanism of pathogenicity and resistance. Moreover, we identified genes that may contribute to the differentiation between species, and tried to build linkages between genetic differences (gene functions and metabolic pathways) and potential pathogenicity. Finally, we uncovered the evolutionary events that may contribute to these variabilities, especially the horizontal gene transfer (HGT) events. Altogether, our study not only provides first insights into genomic features and evolution of the genus *Mobiluncus*, but also has implications for improved understanding of the pathogenic mechanism and putative treatment of this pathogen.

## Materials and Methods

### Genome Data Set

Contigs or scaffolds of the genome sequences for the members of the genus *Mobiluncus* were downloaded from the NCBI genomes FTP site (April 2021)^1^. To avoid bias, genomes with estimated contamination > 5% or completion < 95% were excluded based on CheckM results (Parks et al., 2015). Taxonomy assignment of these genomes was performed by the Genome Taxonomy Database (GTDB) toolkit (Chaumeil et al., 2019) and based on LPSN (List of Prokaryotic names with Standing in Nomenclature) database (Parte, 2018). Contigs of different species were reordered using the Move Contig tool in Mauve software (Darling et al., 2010) against the complete genome of the *M. curtisii* ATCC 43063 and *M. mulieris* DSM 2710, respectively. Pairwise genome alignment was carried out by the lastz program^2^, and the results were visualized using AliTV (Ankenbrand et al., 2017). Pairwise whole genome average nucleotide identity (ANI) values were computed by FastANI (Jain et al., 2018).

### Genome Annotation

All genomes were reannotated using Prokka with default settings (Seemann, 2014). Functional annotation and classification of proteins were performed by sequence comparison using DIAMOND BLASTP (*E*-value 1e-05, coverage 0.5, and identity 40%) (Buchfink et al., 2015) against the recently updated Clusters of Orthologous Group (COG) database (Galperin et al., 2021). KEGG Automated Annotation Server (KAAS) (Moriya et al., 2007) was used for pathway mapping of species-specific genes. Insertion sequences (ISs) were identified by BLASTN against the ISFinder database (*E*-value 1e-05) (Siguier et al., 2006). The prediction of clustered regularly interspaced short palindromic repeat (CRISPR) in the genome was assessed by the CRISPRCasFinder tool (Couvin et al., 2018), and only CRISPRs classified with evidence levels 3 and 4 were considered. Potential ARGs and putative virulence factors (VFs) encoded in genomes were identified through BLASTP searches of the Comprehensive Antibiotic Resistance Database (CARD) (Alcock et al., 2020) and the Virulence Factors Database (VFDB) (Liu et al., 2019), respectively. The most differentiating COG entries between *M. curtisii* and *M. mulieris* were determined by SIMPER analysis by using “simper” function of the vegan R package^3^. “ordinate” function in the phyloseq R package (McMurdie and Holmes, 2013) was used to determine the variation in the functional profiles of HGT genes with non-metric multidimensional scaling (NMDS) analysis on BrayCurtis dissimilarity matrices. Pairwise comparisons of species-specific genomic regions were visualized by EasyFig software (Sullivan et al., 2011).

### Pan-Genome and Phylogenomic Analyses

Homologous gene families were calculated using GET_HOMOLOGUES (Contreras-Moreira and Vinuesa, 2013) with the OrthoMCL clustering algorithm, and cloud, shell, and (soft-) core pangenome components were also derived. Pan-genome statistics were computed by PanGP (Zhao et al., 2014). Marker-based phylogenetic tree of the genus *Mobiluncus* was constructed by the GET_PHYLOMARKERS pipelines (Vinuesa et al., 2018) running in default mode based on nucleotide sequences of 329 high-quality marker genes. In addition. an absence/presence (0/1) matrix of dispensable genes was built according to GET_HOMOLOGUES results, and was subjected to R package pvclust (Suzuki and Shimodaira, 2006) for hierarchical clustering analysis with 1,000 bootstrap replicates with two types of *p*-values: AU (approximately unbiased) *p*-value and BP (bootstrap probability) value.

### Identification of Potential Horizontal Genes

In order to predict the gain and loss of each homologous gene family across ancestral nodes during the evolution of *Mobiluncus*, the pangenome matrix and the rooted phylogenomic tree were used as inputs for COUNT software (Csûrös, 2010) to calculate posterior probabilities (cut-off was set at 70%). We also used HGTector software (Zhu et al., 2014) to detect genes in each genome that were potentially acquired through HGT. During this process, quality cutoffs for DIAMOND BLASTP results were *E*-value ≤ 1e-05, sequence identity ≥ 50%, and coverage of query sequence ≥ 50%. *Mobiluncus* (rank, genus; taxon identifier 2050) was set as the *self* group, and *Actinomycetaceae* (rank, family; taxon identifier 2049) was set as the *close* group.

## Results

### Phylogeny and Genome Overview of the Genus *Mobiluncus*

**FIGURE 1:**
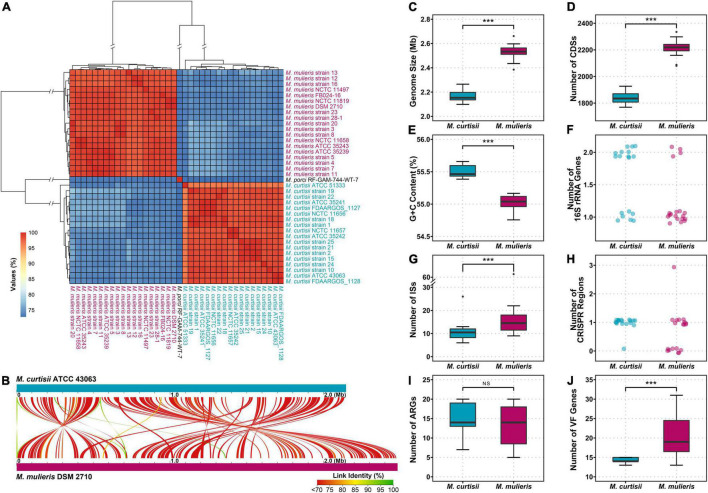
Genomic features of the genus *Mobiluncus*. **(A)** Hierarchical clustering based on ANI values of 38 *Mobiluncus* strains. **(B)** Whole genome alignment of *M. curtisii* and *M. mulieris*. Color denotes percent similarity of links (see legend). **(C–J)** Comparison of genomic characteristics between *M. curtisii* and *M. mulieris*. The boxplot shows the median, and the first and third quartiles as the lower and upper hinges. Outliers are indicated as dots. Asterisks (***) indicate significant differences (significance level of 0.001, Wilcoxon test). NS, not significant; ISs, insertion sequences; CRISPR, clustered regularly interspaced short palindromic repeat; ARGs, antibiotic resistance genes; VF, virulence factor.

**FIGURE 2:**
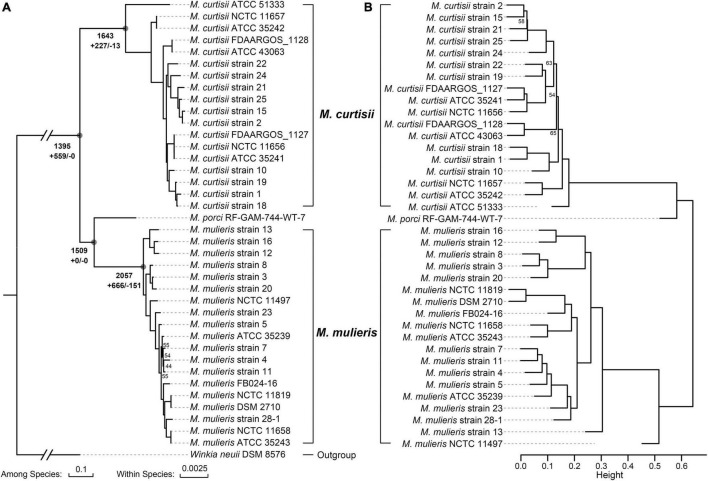
Phylogeny of the genus *Mobiluncus*. **(A)** Maximum-likelihood phylogenomic tree. The tree was constructed based on 329 core genes that could give a well-resolved tree topology without recombination. The total numbers of ancestral orthologous genes present at each of the nodes are shown next to the deep nodes, and the numbers of gene gain (+) and loss (–) events are indicated below. Bootstrap values less than 70% are shown at the nodes. The scale bars indicate 10 and 0.25% sequence divergences among and within species, respectively. **(B)** Hierarchical cluster analysis based on the presence or absence of dispensable genes. Height indicates the dissimilarity between genomes. BP (bootstrap probability) values less than 70% from 1,000 replicates are shown, and all AU (approximately unbiased) *p*-values are > 70%.

All 40 *Mobiluncus* genomes were downloaded from GenBank database (April 2021). Two genomes (strain FDAARGOS_303 and strain NCTC11820) were filtered out as they contained more contamination (8.53 and 11.87%, respectively). Therefore, a total of 38 high-quality *Mobiluncus* genomes were analyzed in this study, including four complete and 34 draft genome sequences (Supplementary Table 1). Based on the similarity observed by GTDB-tk, two valid species are present within the genus, with 18 genomes belonging to *M. curtisii* and 19 genomes belonging to *M. mulieris*. All of these strains were isolated from human vagina (except for two with missing data). In addition, based on LPSN database, strain RF-GAM-744-WT-7 (isolated from pig feces) was classified as *M. porci* (Wylensek et al., 2020). To further elucidate their genetic relatedness, a genome-wide ANI plot was generated (Figure 1A). The intraspecies ANI values of *M. curtisii* and *M. mulieris* were higher than 95.7 and 97.4%, respectively, which exceeded the recommended 95% threshold value for intraspecific prokaryotic strains (Richter and Rossello-Mora, 2009), while the inter-species values were lower than 78.4%. To further evaluate the intra-genus differentiation and evolutionary relationships within the genus, phylogenomic reconstruction was performed based on 329 high-quality phylogenomic marker (core) genes (Figure 2A). The resulting tree shows two major clades corresponding to *M. curtisii* and *M. mulieris* species, and one distinct branch formed by the single strain *M. porci* RF-GAM-744-WT-7 clustering with *M. mulieris*. A hierarchical clustering tree based on the content of dispensable genes showed similar topological structure (Figure 2B), but with *M. porci* RF-GAM-744-WT-7 located on the outskirt of *M. curtisii*. Given the representativeness, the following analyses will focus more on the two validly published BV-associated species, *M. curtisii* and *M. mulieris*.

The characteristics of the genomes studied here are shown in Figures 1B–H and Supplementary Table 1. The average genome size of *M. curtisii* was 2.17 Mbp and was significantly less than *M. mulieris* at 2.53 Mbp (Wilcoxon test, *p* < 0.001). Consequently, *M. curtisii* on average contained fewer protein coding genes at 1,844 compared to *M. mulieris* at 2,213 (Wilcoxon test, *p* < 0.001), and between species showed collinearity with abundant gene arrangement. GC content levels were similar between species, with an average of 55.3%, although *M. curtisii* had a slightly larger GC content than *M. mulieris* (mean, 55.5 and 55.0%, respectively; Wilcoxon test, *p* < 0.001). Strikingly, 11 of 18 *M. curtisii* strains contained two copies of 16S rRNA genes, while only 4 of 19 for *M. mulieris*, and the remaining strains of the two species contained only one copy (Fisher exact test, *p* = 0.0201). The average number of ISs per genome was 10.8 in *M. curtisii* while 17.8 in *M. mulieris*, with *M. mulieris* strain NCTC11497 harboring the greatest number (*n* = 68). CRISPR-Cas system presented in nearly all (17 of 18) *M. curtisii* strains (16 TypeIE and 1 TypeIIC), but only 11 of 19 for *M. mulieris* (4 TypeIE and 7 TypeIIC) (Fisher exact test, *p* = 0.0188). Taken together, these genomic features suggested an apparent genomic divergence between *M. curtisii* and *M. mulieris*.

### Antibiotic Resistance Genes and Potential Virulence Factors of *Mobiluncus*

**FIGURE 3:**
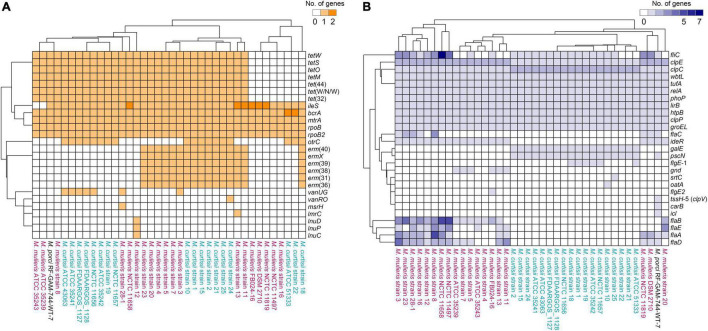
Hierarchically clustered heatmaps of the distributions of the putative antibiotic resistance genes **(A)** and virulence factors **(B)** in *Mobiluncus* genomes.

A total of 26 distinctive putative ARGs were identified. Each genome contained 14.02 ARGs averagely, and no remarkable difference in the total number or profiles of ARGs was found between *M. mulieris* and *M. curtisii* (Figures 1I, 3A). Four ARGs were shared by all *Mobiluncus* strains, including *rpoB* and *rpoB2* (ARO:3004480 and ARO:3000501, respectively; both conferring resistance to rifampicin), *bcrA* (ARO:3002987, conferring bacitracin resistance) and *mtrA* (ARO:3000816, encoding a transcriptional activator of the MtrCDE multidrug efflux pump). Specifically, *otrC* (ARO:3002894) was found to be *M. curtisii*-specific, which encoded a tetracycline resistance efflux pump.

We next investigated the distribution of putative virulence genes. Overall, genomes of *M. mulieris* contained more VFs (mean, 20.42; median, 19) than those of *M. curtisii* (mean, 14.28; median, 14) (Figure 1J), and the profiles of the VFs could largely differentiate the two species (Figure 3B). Ten VFs were shared by all strains, including stress adaptation (*clpC, clpE*, and *clpP*), regulation (*phoP* and *relA*), adherence (*groEL* and *tufA*), secretion (*lirB*), immune evasion (*wbtL*) and others (*htpB*), suggesting they might play key roles in pathogenicity of *Mobiluncus* strains. In addition, six VFs were prevalent in *M. mulieris* but absent in *M. curtisii* strains. Among these, five genes (*flaABCDE*) are associated with bacterial flagellin proteins, which serve as mediators of pathogenicity and host immune responses (Ramos et al., 2004); and the last gene is *ideR*, protein of which has been reported to be the key regulator of VFs and iron homeostasis in *Mycobacterium tuberculosis* (Pandey and Rodriguez, 2014). Moreover, there were two VFs only present in all *M. curtisii* strains: one is *galE*, encoding a UDP-galactose-4-epimerase involved in the biosynthesis of capsular or O-antigen polysaccharide units in many bacterial pathogens (Agarwal et al., 2007; Li et al., 2014); and the other is *pscN*, which encoded ATPase of Type III secretion system as a main VF reported in *Pseudomonas aeruginosa* (Lee and Rietsch, 2015; Ngo et al., 2020).

### Interspecific Pangenome Variation of *Mobiluncus*

**FIGURE 4:**
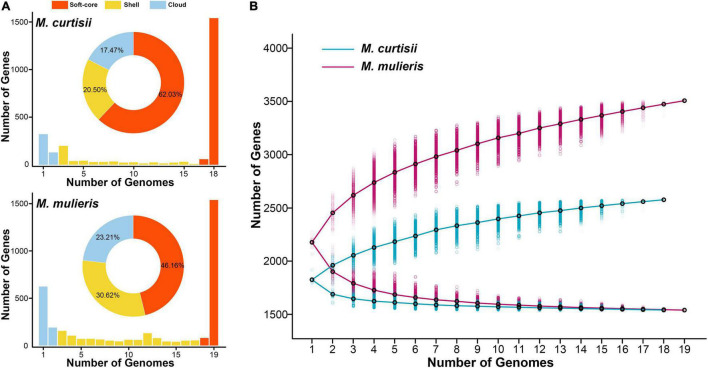
Pangenome summary statistics of *M. curtisii* and *M. mulieris*. **(A)** Histogram distributions of soft-core, shell, and cloud genes. Pie chart displays percentages of each part of the total genes. **(B)** The sizes of pan- and core-genomes in relation to numbers of genomes added into the gene pool.

To explore the interspecific pangenome variation, we characterized the core and pan-genomes of *M. curtisii* and *M. mulieris* separately (Figure 4). The pangenome of *M. curtisii* contained 2,576 genes, whereas that of *M. mulieris* contained 3,507 genes. There was little difference in the core genome size (1,540 and 1,539 genes, respectively; softcore: 1,598 and 1,619 genes, respectively) between the two species. However, it revealed that the pangenome of *M. curtisii* comprised 450 cloud genes (accounting for 17.47% of the total genes) and 528 shell genes (20.50%), much less than those of *M. mulieris* (814 and 23.21%, 1,074 and 30.62%, respectively). According to a power-law regression, both species pangenomes were “open”, with *B*_*pan*_ = 0.19 (*M. curtisii*) and 0.15 (*M. mulieris*). Taken together, both pangenomes appeared to be boundless, while that of the *M. mulieris* was relatively more extensive and heterogeneous.

### Functional Divergence Between *Mobiluncus* Species

**FIGURE 5:**
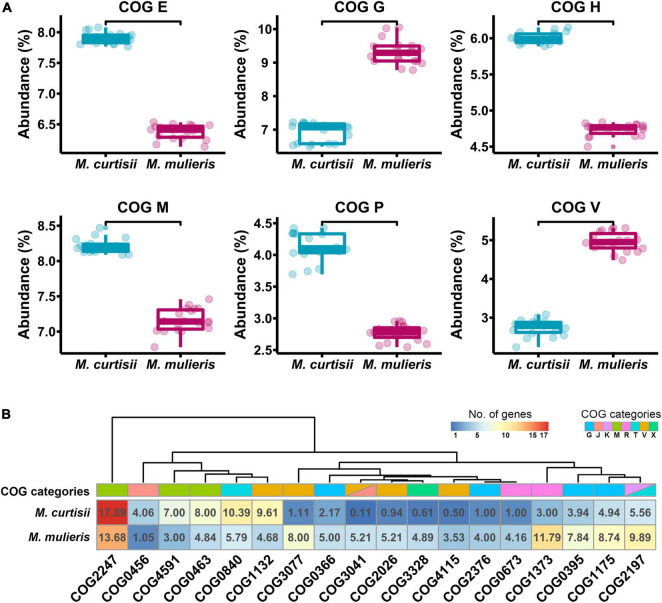
Functional divergences between *M. curtisii* and *M. mulieris*. **(A)** Boxplot of abundance of differential COG categories. All the distributions were significantly different (Wilcoxon test, *p* < 10-5). **(B)** Heatmap of COGs that significantly contributed most to the dissimilarity between *M. curtisii* and *M. mulieris*. Different COG categories are shown in different colors.

To investigate functional differentiation between *M. curtisii* and *M. mulieris*, we first explored the COG functional classification for all genes in each genome (Figure 5A). As a result, *M. curtisii* strains had a higher proportion of genes classified in COG categories E (amino acid transport and metabolism), H (coenzyme transport and metabolism), M (cell wall/membrane/envelope biogenesis) and P (inorganic ion transport and metabolism), while *M. mulieris* strains was significantly enriched for genes classified in COG categories G (carbohydrate transport and metabolism) and V (defense mechanisms). In addition, a total of 18 COGs were detected that significantly contributed most to the dissimilarity between species (SIMPER analysis, > 0.1% contribution, *p* < 0.01) (Figure 5B and Supplementary Table 2), with two-thirds were related to COGs G, V and M. For example, genes associated with cell wall binding/biosynthesis (COG2247 and COG0463) and lipoprotein transport (COG4591) are more abundant in *M. curtisii*, while *M. mulieris* strains contains more genes from RelBE/YafQ-DinJ/Txe-Axe toxin-antitoxin module (COG3077, COG2026, COG4115, and COG3041) and transmembrane proteins of sn-glycerol-3-phosphate transport system (COG0395 and COG1175).

**FIGURE 6:**
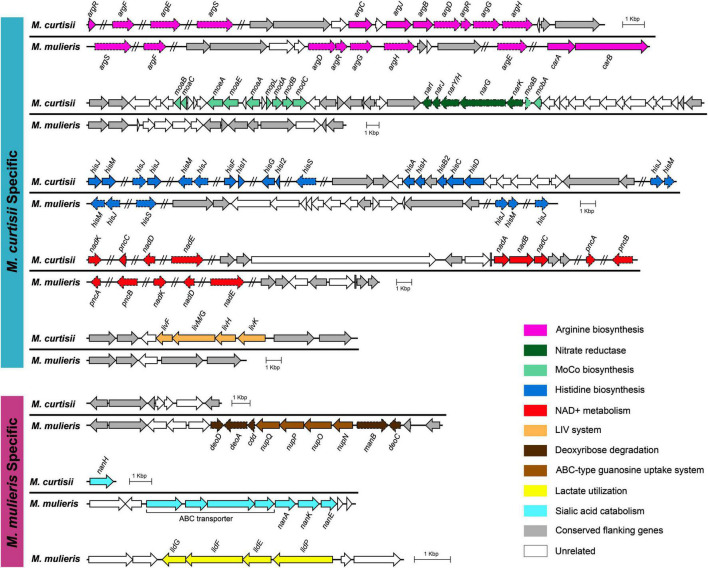
Genetic organizations of the regions containing species-specific genes. Only genes that are universal (> 90%) in one species but absent in the other are determined as species-specific genes. Conserved genomic regions flanking these genes are colored in gray. Non-specific genes are marked with dashed lines. MoCo, molybdenum cofactor.

To further investigate functional differentiation between the species, we explored the species-specific genes that are universal (> 90%) in one species but absent in the other. We found 385 and 429 orthologous genes (OGs) that were specific to *M. curtisii* and *M. mulieris*, respectively (Supplementary Table 3). Some of the OGs were located physically adjacent and clustered into genomic regions (Figure 6), which might perform certain complicated or special roles in extending the metabolic/pathogenic pathways. One such region was composed of several arginine biosynthetic genes. Interestingly, besides operon *argDRGH*, within the region genomes of *M. curtisii* also contained genes of *argCJB*, whereas *M. mulieris* lacked but instead harbored operon *carAB* elsewhere, which has been reported to be necessary for pyrimidine nucleotide and arginine biosynthesis (Han and Turnbough, 1998). Another region contained two pathways, one was involved in the molybdopterin biosynthesis, encoding an ABC-type molybdate transport system and a biosynthetic gene cluster of molybdenum cofactor (MoCo), and another associated with nitrate respiration, encoding a nitrate reductase operon *narKGHJI* (only present in 73.7% *M. curtisii* strains). Moreover, although all genomes had a series of genes related to histidine biosynthesis, another eight *his* genes (*hisF, hisI, hisG, hisA, hisH, hisB, hisC*, and *hisD*) were unique to *M. curtisii* strains. Similarly, operon *nadABC* existed only in *M. curtisii*, enabling them biosynthesize NAD^+^ in both the salvage and the *de novo* pathways. Another *M. curtisii*-specific region contained genes of LIV system, which is responsible for the transport of branched-chain amino acids, such as leucine, isoleucine, and valine (Adams et al., 1990).

We also detected three *M. mulieris*-specific genomic regions. One region was composed of genes of an ABC-type guanosine uptake system NupNOPQ, which inserted in an operon *deoD-deoA-cdd-manB-DeoC*, an unusual *deoCABD*-like operon perhaps serving as deoxynucleotide degradation also found in *Mollicutes* and other pathogens (Christensen et al., 2003; Bizarro and Schuck, 2007). The second was composed of a complete sialic acid catabolic gene cluster *nanAKE*. Interestingly, although *M. curtisii* did not contain the cluster, a sialidase gene (*nanH*) was found to be *M. curtisii*-specific. We also detected a *lldPEFG* operon (orthologs of *lutABC* and *lctP*) in *M. mulieris*, which was implicated in lactate utilization and has been reported to be involved in biofilm formation and pathogenesis in many pathogenic bacteria (Chai et al., 2009; Jiang et al., 2014).

### Gain and Loss of Genes During the Evolution of *Mobiluncus*

**FIGURE 7:**
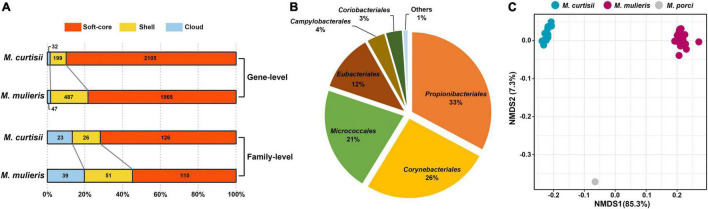
Analysis of predicted transferred genes. **(A)** Distribution of the predicted transferred genes in soft-core, shell, and cloud genes. Numbers of transferred genes and gene families are indicated. **(B)** Pie chart displays percentages of different donors of HGT events at order level. **(C)** Non-metric multidimensional scaling (NMDS) plot of COG entries showing distinct clustering of each species.

To decipher the evolutionary history of the genus *Mobiluncus*, we first assessed the gain and loss events that have occurred of ancestral nodes of species on the phylogenomic tree (Figure 2A). The last common ancestor of the genus *Mobiluncus* was inferred to possess 1,395 gene families. Both of the *M. curtisii* and *M. mulieris* genomes have experienced a massive expansion, with 227 and 666 gene gains have been identified occurred at the divergence of the two species, with only 13 and 151 gene losses, respectively. Next, we further examined the potential horizontal genes in *Mobiluncus* genomes and tracked the potential donor. As a result, a total of 5,000 predicted HGT events were identified, with an average genome containing 131.58 (median, 130) horizontally transferred genes. Interestingly, although *M. mulieris* has a bigger genome than *M. curtisii*, predicted HGT events showed no significant variation between the two species (mean: 131.53 and 129.78, respectively). This may be because the transferred genes of *M. mulieris* comprised more shell and cloud genes (21.4%), while only 9.9% for *M. curtisii*. Correspondingly, 90.1% of the transferred genes of *M. curtisii* were soft-core genes, while only 78.6% for *M. mulieris* (Figure 7A). This content variation also presented at the gene family level.

We next tracked the potential donor of the potential horizontal genes in *Mobiluncus* genomes. Among the 5,000 identified potential HGTs, 57.7% of the donors could be annotated at the phylum level, of which 94.7% were from *Actinobacteria*. The other phyla included *Firmicutes* (4.51%), *Proteobacteria* (0.76%) and *Chloroflexi* (0.035%). In addition, potential donor taxa of 473 genes could be identified at the order level (Figure 7B). The orders *Propionibacteriales, Corynebacteriales*, and *Micrococcales* appeared to be the main donor taxa, accounting for 80% of the total cross-order HGT genes, while order *Eubacteriales* was the most non-actinobacteria donor. We also revealed a different functional profile of putative transferred genes between species. *M. curtisii* acquired a higher proportion of genes classified in COG E (amino acid transport and metabolism), while *M. mulieris* was biased toward COG categories J (translation, ribosomal structure and biogenesis) and V (defense mechanisms) (Supplementary Figure 1). This result was partially consistent with the functional divergence between *Mobiluncus* species described above (Figure 5A). Meanwhile, based on the number of proteins annotated to each COG entries, the three species showed different functional profiles of the HGT genes (PERMANOVA test, *p* = 0.001; Figure 7C), implying HGT contributed to the functional divergence of *Mobiluncus* species.

## Discussion

More than 60 years have passed since BV first described (Gardner and Dukes, 1955), and even now, its etiology and the reason for global prevalence remain unclear (Kenyon et al., 2013; Coudray and Madhivanan, 2020). Common opportunistic bacteria causing BV include *Prevotella, Gardnerella vaginalis* and *Mobiluncus* (Thorsen et al., 1998; Coudray and Madhivanan, 2020), and the abundance of *Mobiluncus* strains always represents a higher Nugent score and a higher possibility to fail therapy (Schwebke and Desmond, 2007; Meltzer et al., 2008). For the first time, in the current study we tried to reveal genomic details of the genus *Mobiluncus*, to gain more insights into the genomic features, VF and ARG profiles, functional repertoire and the evolutionary history of *Mobiluncus* diversification. Such information would provide theoretical foundation for further studies on the pathogenicity, therapy and discrimination of *Mobiluncus* species.

Efforts have been made to distinguish the two main species of *Mobiluncus* on the basis of morphological and biochemical differences, especially on the antigenic profiles (Roberts et al., 1984, 1985; Spiegel, 1987; Zhang et al., 2020). In this study, we performed a robust phylogenomic reconstruction to verify the degree of differentiation among species, emphasizing the genetic differences between *M. curtisii* and *M. mulieris*. We showed that the genome size of *M. mulieris* was relatively larger, with more gene family gains predicted across its evolution and a more open pangenome. This is consistent with the fact that *M. mulieris* strains comprised more ISs but less CRISPR-Cas systems within the genomes, both of which have been reported to play important roles in the bacterial genome instability (Darmon and Leach, 2014; Hatoum-Aslan and Marraffini, 2014). In addition, a genomic investigation on ARG and VF profiles showed four ARGs and ten VFs were found to be prevalent in all *Mobiluncus* strains, while the remaining other genes exhibited sporadic distribution patterns. Moreover, VFs profiles were able to distinguish *M. curtisii* from *M. mulieris*, whereas ARG profiles were not. Correspondingly, previous experimental studies have also revealed significant intra- and inter-species heterogeneity of antimicrobial susceptibility (Spiegel, 1987; Zhang et al., 2020). Also, it should be mentioned that although the role of *Mobiluncus* in the etiology and pathology of BV remains unclear, these two species may exhibit different pathogenicity and distribution during the disease process (Meltzer et al., 2008; Onderdonk et al., 2016; Arries and Ferrieri, 2022), sometimes even contradictory (Schwebke and Lawing, 2001; Salinas et al., 2020). Nevertheless, the VF and ARG profiles revealed in this work may provide guidance for the future treatment of *Mobiluncus* infection.

We also detected a series of metabolic pathways that showed apparent species specificity, most of which have been reported to be associated with virulence and adoption of pathogenic organisms. For example, the role of arginine biosynthesis in virulence has been reported to be crucial for full virulence of *Aspergillus fumigatus* in insects (Dietl et al., 2020), and we have showed that the pathway of arginine biosynthesis in *M. curtisii* and *M. mulieris* was different, perhaps suggesting a different utilizing efficiency. Furthermore, the capabilities of MoCo biosynthesis and nitrate reduction were only found in *M. curtisii* strains, both of which have been implicated in pathogenesis of a number of bacterial infections (Williams et al., 2011; Andreae et al., 2014; Almeida et al., 2017); more *his* genes were found in *M. curtisii* strains, perhaps enabling them capacity of biosynthesis of histidine and a crucial role in metal homeostasis and virulence (Dietl et al., 2016); and the extra NAD^+^ *de novo* biosynthesis pathway in *M. curtisii* could also enhance their virulence during host infection (Dom and Haesebrouck, 1992; Wang et al., 2019). On the contrary, two virulence-associated gene clusters, including genes associated with ABC-type guanosine uptake system NupNOPQ and lactate utilization, were only present in *M. mulieris*. Another noteworthy was the *nan* gene cluster for sialic acid catabolism (SAC). With these genes *M. mulieris* strains were more likely to consume host sialic acids as carbon source but could not cleave terminal Neu5Ac residues from host glycoconjugates (lacking *nanH*), whereas *M. curtisii* did just the opposite. This pattern perhaps implicated a cooperation between closely related species. However, this cooperation relationship seems not to be strictly necessary, as women with BV could harbor both or either of the two species (Holst, 1990). SAC associated genes have been detected in many BV-associated bacteria, which could enhance the pathogenicity of organisms by allowing easier invasion and destruction of tissues (Hardy et al., 2017; Jones, 2019; Li and Huang, 2022). These results could reinforce further discrimination of *Mobiluncus* species, perhaps by providing a simple and fast approach for identifying *M. curtisii* and *M. mulieris* using PCR or culture experiments, and in addition might facilitate the development of novel strategies to detect and prevent *Mobiluncus* infection of BV.

HGT is known to have great, perhaps the most conspicuous, impacts on bacterial diversity and speciation, especially for clinical microorganisms, where acquisition of foreign genes is crucial for pathogenicity (Smillie et al., 2011; Diard and Hardt, 2017; Arnold et al., 2021). In this study, we have used several methods to evaluate the HGT events. Firstly, both of the two *Mobiluncus* species have an open pangenome, which could be considered as an indicator of high HGT rates (Medini et al., 2005; Tettelin et al., 2008). Then, we reconstructed the evolutionary history of the genus. As expected, gene families undergoing gain events at ancestral nodes of species outnumbered those that experienced loss events. Therefore, differences in metabolism and pathogenicity between species have emerged. Finally, by using a BLAST-based HGT detection approach, we found that more than 5% of genes in each strain have suffered transfer events, and these genes perhaps further promoted the functional divergence between *Mobiluncus* species. Interestingly, no significant correlation between genome size and HGT frequency was observed, which probably means strains of *M. mulieris*, compared to *M. curtisii*, tend to acquire more dispensable genes, or meanwhile have suffered more gene loss events. Most of the transferred genes originated within the *Actinobacteria* phylum, with more from members of orders *Propionibacteriales, Corynebacteriales*, and *Micrococcales.* These orders have been reported to include many pathogenic species that could cause devastating diseases in humans and animals (Barka et al., 2016; Park et al., 2019), and also include microorganisms that are also present in the human vagina (Funke et al., 1997; Aleshkin et al., 2006; de Figueiredo Leite et al., 2010; Okoli et al., 2019). Taken together, these findings suggested that genome dynamic, mediated by gene gain and loss, might be an important strategy for *Mobiluncus* species diversification, host adaptation and pathogenicity.

Collectively, the present study largely extends the understanding of the genomic features, virulence and antibiotic resistance profiling, and evolution of the genus *Mobiluncus*. Our results also highlight the difference between *M. curtisii* and *M. mulieris*, providing more clues for distinguishing of the two species. Nevertheless, more experimental evidences are needed to verify these differences. Fully understanding the pathogenic potential of *Mobiluncus* strains remains a complex task with much to be explored in the future.

## Data Availability Statement

The genomes analyzed in this study are all available in NCBI GenBank database with the accession numbers listed in Supplementary Table 1.

## Author Contributions

YL designed the study, performed bioinformatic analyses, and wrote the draft manuscript. JL contributed to the conception of the study. YW and JL interpreted, discussed the results, and revised the manuscript. All authors contributed to manuscript revision and approved the submitted version.

## Conflict of Interest

The authors declare that the research was conducted in the absence of any commercial or financial relationships that could be construed as a potential conflict of interest.

## Publisher’s Note

All claims expressed in this article are solely those of the authors and do not necessarily represent those of their affiliated organizations, or those of the publisher, the editors and the reviewers. Any product that may be evaluated in this article, or claim that may be made by its manufacturer, is not guaranteed or endorsed by the publisher.

## Footnotes

1
https://www.ncbi.nlm.nih.gov/genomes

2
https://github.com/lastz/lastz

3
https://github.com/vegandevs/vegan
