# Supplementary figures and images for: Genomic Insights Into the Interspecific Diversity and Evolution of Mobiluncus, a Pathogen Associated With Bacterial Vaginosis
Source: Front Microbiol. 2022 Jul 5;13:939406. doi: 10.3389/fmicb.2022.939406 (PMC9294530; doi:10.3389/fmicb.2022.939406)

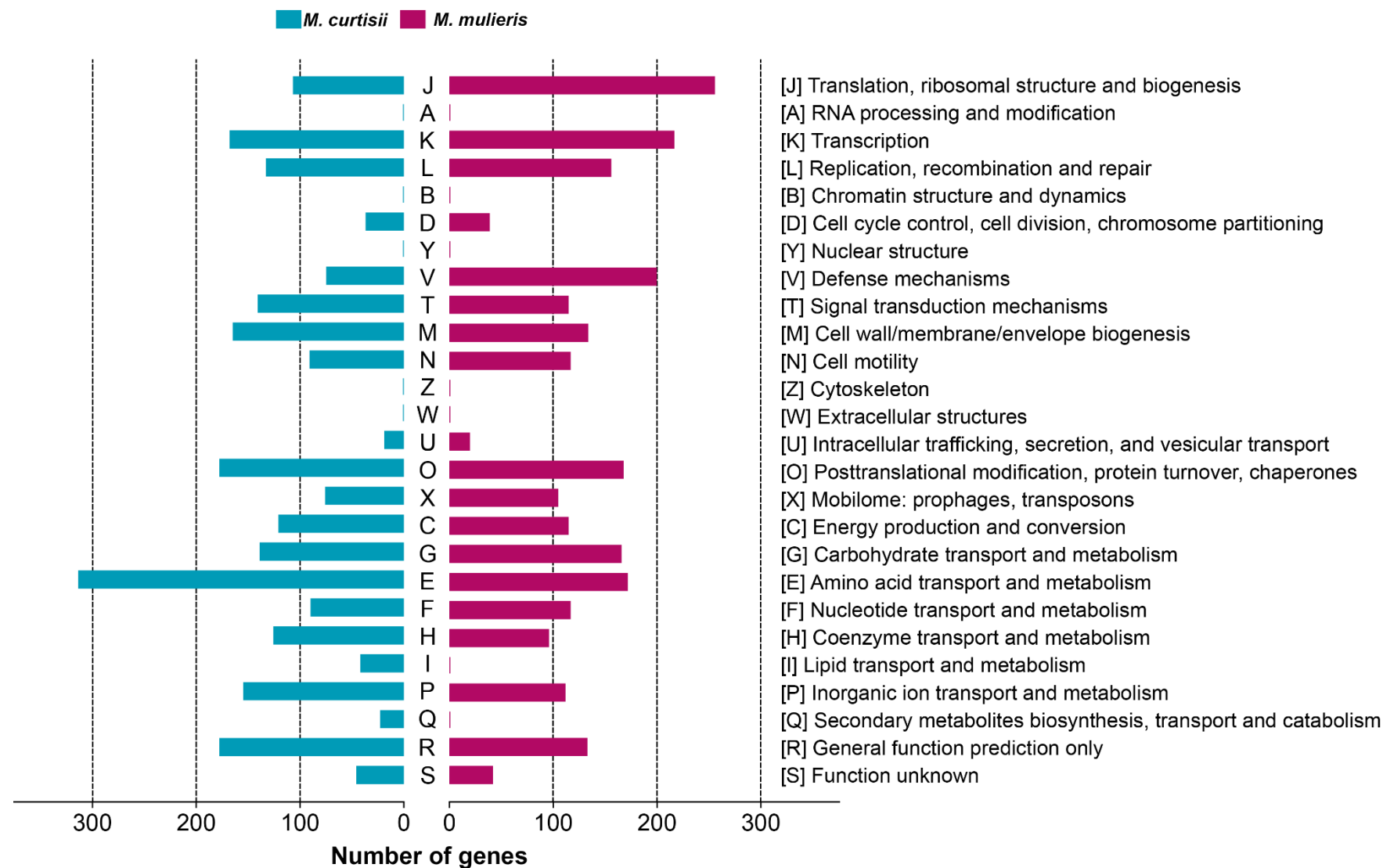

**Supplementary Figure 1. Distribution of HGT genes in different COG categories.**

Supplement: Supplementary file 1 [file Presentation_1.PDF]
